# Educator’s role in preventing ageism

**DOI:** 10.1371/journal.pone.0313137

**Published:** 2024-11-04

**Authors:** Špela Mihevc, Anja Grušovnik Mušič, Danica Rotar Pavlič

**Affiliations:** 1 Department of Occupational Therapy, Faculty of Health Sciences, University of Ljubljana, Ljubljana, Slovenia; 2 Elderly Home Škofljica, Škofljica, Slovenia; 3 Department of Family Medicine, Faculty of Medicine, University of Ljubljana, Ljubljana, Slovenia; Instituto Nacional de Geriatria, MEXICO

## Abstract

**Aims:**

This study aims to contribute to a better understanding of the educator’s role in imparting knowledge to healthcare students regarding ageism in Slovenia.

**Methods:**

Educators in Slovenian secondary nursing schools and in medical and health science faculties were asked to evaluate their practical experience in working with older people, their knowledge of gerontology and working with older people, and their attitude toward working with older people using an online questionnaire. They were also asked to rate their opinion about ageism on a Likert scale, along with reasons for ageism, skills that would help reduce ageism, their opinion about trainees’ attitudes toward older people, and their assessment of certain facts about planning training. The reliability of the questionnaire was tested using Cronbach’s alpha. The Kruskal‒Wallis test, Mann‒Whitney *U* test, and independent samples *t*-test were used to determine differences between the groups.

**Results:**

The participants fully agree that the knowledge imparted could contribute to reducing age discrimination among healthcare students. Those that responded that their gerontological knowledge is good had significantly higher ranked responses regarding the reasons for ageism. On average, they agreed with the statements about planning their teaching activities and facilitating the acquisition of knowledge.

**Conclusions:**

Ageism is a challenge for modern society that requires a comprehensive approach to prevent and combat this form of discrimination. Awareness-raising, education, and policy change can create a fairer and more respectful society for all generations. Educators are insufficiently aware of their role in preventing ageism. Practitioners recognize it when they work directly with students. There are opportunities to update curricula and teaching methods.

## Introduction

The trend toward an ageing population requires a critical examination of the situation in healthcare facilities with regard to the way healthcare staff treat older people. The World Health Organization published its *World Report on Ageism* on 18 March 2021 [1]. Addressing ageism is one of the four action areas of the Decade of Healthy Ageing 2021–2030. Ageism is a major health determinant that is recognised as a major threat to healthy ageing and an important public health challenge [2, 3]. A quantitative exploratory study concluded that it is essential to further study attitudes toward working with older people and to take improvement measures based on the research findings because the need for healthcare for older people will increase [4]. The quality of healthcare services that older people receive is directly related to the perceptions, knowledge, and skills of healthcare professionals [5, 6]. There are various definitions of ageism, but what they all have in common is a dislike of older people and their personal or social undervaluation, disregard, neglect, and exclusion (marginalisation), as well as a personal dislike of ageing, old age, and all that is associated with it. Ageism can take the form of stereotypes that reflect the way people think about older people, prejudice that reflects perceptions, and discrimination that reflects attitudes toward older people [1].

Based on a literature review conducted by Iversen et al. [7], ageism can be conscious or unconscious, and it can be reflected at the micro (individual), meso (social networks), or macro (institutional and cultural) levels [2, 8]. Discrimination against older people is not a new phenomenon, but it has increased in recent years, including in Slovenian society, where discriminatory attitudes toward older people are on the rise against the backdrop of a worsening economic situation, the growing number of older people, and the resulting decline in the working population [9].

Research shows that ageism also occurs in health and social care settings, but there is insufficient research on the prevalence of ageism among health professionals [10]. Older people are not aware of ageism because they are accustomed to the authoritarian attitudes that used to prevail in healthcare institutions in the past or because ageism is subtle [11, 12]. Older people are not aware of ageism because they can direct this prejudice towards themselves. The process of aging can be viewed unfavorably by some people, who view it pessimistically. Consequently, age stereotypes are fixed beliefs that overgeneralize the characteristics, attributes, and behaviors held in society and become somehow socially acceptable [13]. Ageism is a form of discrimination that justifies and perpetuates inequalities in the health treatment of individuals and groups of various ages and reduces opportunities for older people in many areas of life [3, 14, 15]. How health professionals view the health of older people is important because this has an impact on treatment outcomes and patients’ quality of life [16]. Several studies have found a link between increasing age and decreasing healthcare quality [9, 14, 17–20]. Harmful stereotypes, prejudice, and ageism affect healthcare. It is not known to what extent the ideology of stigmatisation is entrenched in healthcare processes and how it affects professional values [21].

More data and evidence are needed to inform formal and informal educational interventions [22]. The results of the only Slovenian survey carried out on the subject to date [23] confirm the presence of ageism in clinical settings, with more than half of respondents having experienced at least one discriminatory event. Because ageism has a negative impact on attitudes toward older people and the quality of their care, professional communication with older people needs to be included in the training of all health professional profiles [4, 6, 22]. Improved knowledge and skills of health professionals working with older people can significantly contribute to improving the quality of care and lead to a reduction in negative attitudes toward working with older people [5]. Appropriate attitudes, specialised skills, and understanding the role of interdisciplinary collaboration can help improve the care of older people and eliminate stereotypes [22, 24].

Assessing (measuring and identifying) ageism is important for health professionals and legislation to develop and implement strategies to prevent or reduce ageism [18, 25].

Based on the literature review, it was concluded that education and improving professional communication is a very effective and important tool for preventing ageism. Research shows that knowledge about ageing can reduce ageism in health professions and improve care for older people. Given the increasing number of older people and the presence of ageism, educating future health professionals can be a challenge for educators. Educational interventions, such as intergenerational learning programs and simulation-based learning have been shown to promote more positive and inclusive views of aging across multiple contexts (32).

Research findings (8, 12, 15, 26, 28) demonstrate the fact that younger generations find it more difficult or choose not to work with older people. This trend reflects the state of society and encourages collaboration between policy makers, professionals, academics and the community.

The Slovenian education system is organised into several levels. The first-, second-, and third-cycle (bachelor’s, master’s, and doctoral) programmes are offered by public or private universities and individual higher education institutions, which are supervised by the Nakvis agency, which provides for the development and operation of the quality assurance system in Slovenian higher education in accordance with European and global development orientations. This study included higher education institutions and academic programmes that demonstrate the appropriate quality of education through accreditation granted by the agency.

For the purpose of this study study we examined the extent to which topics related to ageism are included in the curricula for the education of doctors, occupational therapists, physiotherapists and nurses in Slovenia. We used a qualitative research design with an explanatory method in combination with elements of content analysis. We identified several content components in the gerontology curricula that can be indirectly linked to ageism. Apart from the two compulsory subjects at the first level and the two elective subjects at the second level of education, ageing was not included.

## Aims

This study aims to contribute to a better understanding of the educator’s role in imparting knowledge to healthcare students regarding ageism.

## Methods

This research was carried out as part of a doctoral dissertation at the University of Ljubljana’s Faculty of Medicine. The Medical Ethics Committee gave its approval for the study on 13 April 2023 (ethics review no. 0120-74/2023/3). The questionnaire was developed as part of the dissertation *Dimensions of Ageism–A Qualitative Exploration among Older Adults and Healthcare Workers*. In the address of the participant there was no informed consent, because the privacy policy itself in the online survey 1KA (an open-source application providing online survey services)., which has the general rule that the authors of the survey may not obtain any information about the participants without their prior knowledge and consent. The responsible persons of 1KA are aware of the importance of data protection and act in accordance with the Personal Data Protection Act and Regulation (EU) 2016/679 of the European Parliament and of the Council on the protection of natural persons with regard to the processing of personal data and on the free movement of such data, and repealing General Data Protection Regulation.

The literature review revealed an important theme regarding the role of educators in imparting knowledge to future healthcare workers to prevent ageism in healthcare organisations. To gain a better understanding of the topic, five sets of questions were developed. To achieve more analytical objective we wanted to find out the difference between attitude, knowledge and experience between the 5 sets of questions.

Participants answered the questions in these sets using a five-point Likert scale (1 = strongly disagree, 5 = strongly agree). The first set (S1) contained 10 statements about ageism, the second set (S2) contained seven statements about the reasons for ageism, the third set (S3) included nine statements about skills that would help reduce ageism, the fourth set (S4) featured six statements about the trainees’ attitudes toward older people and working with them, and the fifth set (S5) contained six statements about the trainees’ educational work**.** Those are dependent variables (S1-S5). This was followed by questions on whether the participants had practical experience in working with older people, their attitudes toward working with older people, and how they would rate their knowledge of gerontology and working with older people. Finally, they were asked to indicate their job title, their level of employment, the region where they were employed, their years of service, their age, and their sex. Those are independent variables.

The paper-based questionnaires were initially given to 10 participants directly engaged in training healthcare students. After completing the questionnaire, they were briefly interviewed to establish whether they had understood the questionnaire and whether they had had any difficulties completing it. We adapted some of the questions slightly to make them easier to understand.

The data were then collected between 7 November 2023 and 21 February 2024 using an online survey on 1KA. The questionnaire was e-mailed to all 19 secondary nursing schools, health science faculties, and medical faculties in Slovenia via publicly available contact information. Clinical mentors were also invited to participate in the survey and contacted by the national coordinators for clinical education. An invitation to complete the questionnaire was sent to the same addressees three times with a request for forwarding.

IBM SPSS® Statistics software, version 26.0, was used for the statistical analysis of the data. Descriptive statistics were used to calculate frequencies, mean values, and standard deviations. Cronbach’s alpha was used to determine the internal consistency of the questionnaire across the Likert scale items, and it was calculated separately for each set of questions. For each set, the mean of the responses was calculated with the standard deviation (Table 1). The groups were then compared with questions on practical experience of working with older people, attitudes toward working with older people, and assessment of their knowledge of gerontology and working with older people to determine differences between the groups.

**Table 1:** Reliability of individual questionnaire items, mean value of clusters, and differences between academics and practitioners.

Sets (*n* = 88)	Reliability^a^: *n* items, α	Mean of cluster, *SD*^b^	Mean of academics, *SD*^b^, mean rank	Mean of practitioners, *SD*^b^, mean rank	Differences between academics and practitioners^c^	Questions^d^
1: Statements about ageism	10,0.716	3.748 ± 0.481	3.734 ± 0.451, 43.33	3,762 ± 0.511, 45.62	0.672	1‒10
2: Reasons for ageism	7,0.678	3.754 ± 0.527	3.784 ± 0.465, 45.60	3.727 ± 0.584, 43.44	0.690	11‒17
3: Skills to reduce ageism	9,0.869	4.369 ± 0.508	4.366 ± 0.406, 42.88	4.372 ± 0.593, 46.04	0.558	18‒26
4: Ageism among trainees	6, 0.877	2.925 ± 0.529	2.813 ± 0.483, 38.05	3.029 ± 0.554, 49.56	**0.030**	27‒32
5: Educational work	6,0.835	3.913 ± 0.670	3.920 ± 0.700, 44.00	3.907 ± 0.648, 44.00	1.000	33‒38

^a^Cronbach’s alpha

^b^Standard deviation

^c^Asymp. sig. (two-tailed) Mann–Whitney *U* test

^d^Supplementary material S1 Fig.

The Shapiro‒Wilk test was used to check the normality of data distribution. If the data were not normally distributed, two non-parametric tests were used for further analysis: the Kruskal‒Wallis test for differences between more than two groups and the Mann‒Whitney *U* test for differences between two groups. If a result proved significant on the Kruskal‒Wallis test (*p* < 0.05), the Dunn‒Bonferroni post-hoc method was used. If the data distribution was normal, the parametric *t*-test (independent samples test) was used.

## Results

A total of 146 individuals took part in the survey. After eliminating the surveys with missing responses, 88 questionnaires were included in the analysis. The decline in responses was systematic from the first to the last question, and it was greatest in the middle of the survey for the question "Existence of ageism ". This part of the survey required a particularly critical look at one’s own work.

The average age of the participants was 48 (± 10.6) years. Their average length of service was 23.6 (± 10.9) years. Among the participants, 86.4% (*n* = 76) were female and 13.6% (*n* = 12) were male. Most of them taught in vocational bachelor’s programmes (55%) and academic bachelor’s programmes (17%), followed by master’s programmes (10%), doctoral programmes (9%), individual master’s programmes (5%), and secondary vocational education (4%).

When asked to rate their own knowledge of gerontology and working with older adults, the participants reported that their knowledge was good on average (mean = 3.84 ± 0.709). On average, they reported that their attitude toward working with older people was good or that they were interested in working with them (mean = 4.10 ± 788). Among all participants, 93.2% had practical experience of working with older people and 6.8% had no experience at all. Among the respondents, 51.1% were employed in practice-oriented jobs (mentors, placement organisers, and placement teachers), and the remaining 48.8% were academics.

The results of Cronbach’s alpha show that all five clusters have a satisfactory internal consistency (Cronbach’s alpha = 0.678–0.835). We named the clusters S1 to S5, as shown in Table 1. The mean scores show that, on average, participants in area 1 agree with the statements about age discrimination as well as with the reasons for age discrimination. They fully agree that the knowledge imparted could help reduce age discrimination. On average, they neither agree nor disagree with the statements that trainees have discriminatory or stereotypical attitudes and prejudices toward older people and working with them. On average, they agree with the statements about planning their teaching activities and facilitating the acquisition of knowledge. We tested the difference between academics and practitioners. A significant difference between the two groups was only found for S4 (*p* = 0.03), which concerns the participants’ opinion about age discrimination among trainees. Compared to academics, practitioners are of the opinion that there is more age discrimination among trainees. The biggest difference was in the opinion that trainees stereotype and have prejudices against older people. There were no differences between academics and practitioners in the assessment of knowledge and attitude to work (Table 1).

The individual strands were compared based on the variables of knowledge assessment, attitude toward working with older people, and practical experience with older people. First, the Shapiro‒Wilk test was used to check whether the data were normally distributed. For most variables, the distribution was not normal, and so non-parametric tests were used. The significance values are listed in Table 2. A normal distribution was only established for the variable S1 within the two groups, which refers to practical experience in working with older people, and so a parametric *t*-test was used in this case. Significant values below 0.05 are provided in bold in the table to show that there are differences between the groups (Table 2).

**Table 2:** Differences between groups of independent variables and dependent variables.

Variables, *n*	Group	S1	S2	S3	S4	S5
1. Knowledge of gerontology, 88	Very poor	0.105^a^	**0.017** ^a^	0.467^a^	**0.002** ^a^	**0.009** ^a^
	Poor					
	Neither					
	Good					
	Very good					
2. Attitude toward working with older people, 88	Not interested at all	0.057^a^	0.218^a^	**0.007** ^a^	0.506^a^	**0.004** ^a^
	Not interested					
	Neither					
	Interested					
	Very interested					
3. Practical experience in working with older people, 87	Yes	0.391^c^	0.434^b^	0.610^b^	0.771^b^	**0.013** ^b^
	No					

^a^Asymp. Sig. Kruskal–Wallis *H* test

^b^Asymp. Sig. (two-tailed) Mann–Whitney *U* test

^c^Sig. (two-tailed) independent samples *t*-test, S1 = Statements about ageism, S2 = Reasons for ageism, S3 = Skills to reduce ageism, S4 = Ageism among trainees, S5 = Educational work

If the non-parametric Kruskal‒Wallis test revealed statistically significant differences, we also performed a Dunn‒Bonferroni post-hoc analysis of the data, which showed exactly which groups were different. The results are presented in terms of the mean rank and significance. Table 3 shows the results of the significant Mann‒Whitney *U*-test with the mean rank.

**Table 3:** Differences between groups’ knowledge, attitude toward working, and practical experience.

Comparison	Group	Mean rank	Sig
S2 and S1. Knowledge of gerontology	Good	50.97	0.017^a^
	Neither	29.33	
S4 and S1. Knowledge of gerontology	Good	49.01	0.026^a^
	Neither	28.81	
S5 and S1. Knowledge of gerontology	Very good	62.23	0.024^a^
	Neither	32.71	
S3 and S2. Attitude toward working with older people	Very interested	58.57	0.010^a^
	Interested	38.53	
S5 and S2. Attitude toward working with older people	Very interested	59.59	0.005^a^
	Interested	38.36	
	Very interested	59.59	0.026^a^
	Neither	32.55	
S5 and S3. Practical experience working with older people	Yes	45.83	0.013^b^
	No	19.33	

^a^Adj. Sig. Bonferroni correction

^b^Asymp. Sig. (two-tailed) Mann–Whitney *U* test, S1 = Statements about ageism, S2 = Reasons for ageism, S3 = Skills to reduce ageism, S4 = Ageism among trainees, S5 = Educational work

As can be seen in Table 3, there is a significant difference between the participants that answered that their knowledge is good and those that reported it was neither good nor bad. Those that responded that their knowledge was good had significantly higher ranked responses regarding the reasons for ageism (*p* = 0.017) and responses about the presence of ageism among trainees (*p* = 0.026). Participants that responded that their knowledge was very good rated the answers about planning their curricula and the process of working with trainees higher than the participants whose knowledge was neither good nor bad (*p* = 0.024).

There is also a difference between the groups that responded that they were very interested in working with older people and those that were not. Those that responded that they were very interested in working with older people rated the answers about the importance of skills to avoid ageism higher (*p* = 0.010). They also rated responses about the curriculum design and work process higher than those that were not interested in working with older people (*p* = 0.005), and there was also a difference between the groups that were very interested and not interested in working with older people (*p* = 0.026).

Those that had practical experience of working with older people rated the curriculum design and learning process responses higher than those that had no experience (*p* = 0.013).

The individual questions in S2 (reasons for ageism) were analysed using the Kruskal‒Wallis test, which showed that respondents with higher knowledge scores indicated that the most important reasons for ageism were the personal (*p* = 0.008) and social underestimation (*p* = 0.012) of older people and the marginalisation of older people in society (*p* = 0.019).

## Discussion

Considering that combating ageism is one of the four action areas of the Decade of Healthy Ageing, educators have the responsibility to implement and analyse specific actions to reduce ageism among healthcare students to ensure high quality of care provided to the ageing [26]. In an aging society, medical education needs to focus more on activities aimed at displaying positive attitudes toward older people. To develop proper contact with older people, the solution may be to influence modifiable factors, especially the correct education of future healthcare workers. Well-trained healthcare staff is one of the most critical challenges of healthcare systems worldwide, and it seems to be a massive challenge for schools educating future healthcare professionals [27]. San-Martin-Gamboa et al. suggest that an intervention combining ageing education with clinical practice can significantly reduce negative stereotypes and prejudices about ageing [26]. In our study, we invited educators to share their opinions and experiences, and it was found that participants that ranked their knowledge of gerontology and working with older people as very good and good had better knowledge and understanding of the causes of ageism, they perceived ageism to be more prevalent among trainees, and they were better able to plan and adapt their teaching and curricula accordingly. Participants in the study that are interested and very interested in working with older people have better knowledge and understanding of the reasons why ageism occurs, and so they plan and adapt their teaching and curricula accordingly. Participants with practical experience of working with older people can plan and adapt their teaching and curricula better. Practitioners perceive that there is more stereotypical thinking about older people and prejudice against older people among trainees compared to academics. The findings of a systematic review and meta-analysis carried out by Burnes et al. suggest that feasible strategies involving education and intergenerational contact can serve as the basis for effective interventions to reduce ageism [28]. Samra et al. indicated that the quality of previous relationships with older people was linked to attitudes, with good quality relationships with older people related to positive outcomes, motivation, and preference for working with older people [29]. Mendez et al. highlighted that educational interventions are a powerful tool to diminish prejudices and misconceptions of ageism in future healthcare professionals [30]. The findings of their study show that training and engaging students early in their programmes are of great value in creating awareness and changing prejudices in ageist attitudes among healthcare students. Education is the most powerful tool for changing misconceptions about ageing. In the long term, this may be the key to eliminating ageism.

Ageism in healthcare is a threat to older people’s dignity, rights, health, and well-being. Creating change by combating ageism at both the micro and macro levels is possible and should be a priority, given that the world’s population is ageing. Intergenerational activities, educational programmes, policy changes, and practice reform can transform the current healthcare culture and facilitate the provision of ethical and equitable whole-person care to older people. It takes willingness to try [31].

Practitioners notice ageism when working with students, academics do not. Therefore, it is necessary to address them in this regard. It has been noted that this is the reason why they do not address it in their pedagogical work.

## Strengths and limitations

A limitation of this study is the relatively small sample size of the various groups of professionals that train healthcare students. Because the participants were only asked to provide their occupation at the end of the survey, we have no information on which occupations were most likely to not be covered during the survey. In fact, the last two sets of questions (curricula and incidence of age discrimination among trainees) were the ones most frequently omitted. Another limitation of the study is that we have no data on the final number of questionnaires sent out due to forwarding by the participants.

One of the benefits of the survey is that the questionnaire proved to be reliable. This is the first survey that can help in understanding the educator’s role in imparting knowledge about ageism to healthcare students in Slovenia.

## Conclusion

In recent decades, the study of ageism has increased due to the growing old population. Ageism and other attitudes of healthcare professionals can negatively impact care for older adults. It is essential to implement actions to reduce ageism among healthcare students. Education is one tool ‒ perhaps the most powerful one ‒ to combat ageism. We have no specific educational interventions for students. The educators are the first mediators of information and knowledge. Knowledge can have a positive impact on attitudes towards working with older people.

Policymakers, managers, and educators must continue developing education for healthcare professionals engaged in older people’s care. Students need to be supported to meet the complex needs of older people. It is of paramount importance that healthcare students become qualified health professionals that have not only the willingness, but also the knowledge, skill, attitudes, and value-based competence to provide high-quality care to an increasingly ageing society. Teaching must be adapted to the characteristics of the younger generation to understand the incidence and causes of ageism. Knowledge needs to be strengthened and attitudes toward working with older people need to be improved by increasing the opportunities for intergenerational cooperation.

Recommendations for further research would aim to find out how educators specifically address ageism in their lectures and what specific educational interventions they use for students.

## Supporting information

S1 Fig Survey. (PDF)

S2 Fig Raw data. (ODS)

S3 Fig Values data. (PDF)

## References

[1] World Health Organization. Global report on ageism, https://www.who.int/teams/social-determinants-of-health/demographic-change-and-healthy-ageing/combatting-ageism/global-report-on-ageism (2021, accessed 25 April 2023).

[2] Marques S, Mariano J, Mendonça J, et al. Determinants of ageism against older adults: a systematic review. Int J Environ Res Public Health 2022; 17(7): 2560. 10.3390/ijerph17072560 PMC7178234 32276489

[3] Ayalon L and Cohn-Schwartz E. The relationship between perceived age discrimination in the healthcare system and health: an examination of a multipath model in a national sample of Israelis over the age of 50. J Aging Health 2022; 34(4–5): 684–692. doi: 10.1177/08982643211058025 34866449

[4] Skela Savič B and Hvalič Touzery S. Znanje, odnos in dojemanje dela s starejšimi osebami pri zaposlenih v zdravstveni negi in oskrbi v domovih za starejše: eksplorativna raziskava. Obzor Zdr N 2020; 54(1): 38–51.

[5] Gholamzadeh S, Shaygan M, Naderi Z, et al. Age discrimination perceived by hospitalized older adult patients in Iran: a qualitative study. Health Promot Perspect 2022; 12(1): 45–55. doi: 10.34172/hpp.2022.07 35854844 PMC9277281

[6] Shroyen S, Adam S, Jerusalem G, et al. Ageism and its clinical impact in oncogeriatry: state of knowledge and therapeutic leads. Clin Interv Aging 2015; 10: 117–125. doi: 10.2147/CIA.S70942 25678781 PMC4317143

[7] Iversen TN, Larsen L and Solem PE. A conceptual analysis of ageism. Nord Psychol 2012; 61(3), 4–22.

[8] Wyman MF, Shiovitz Ezra S and Bengel JB. Ageism in the healthcare system: providers, patients and systems. In: Ayalon L and Tech-Römer C (eds) Contemporary perspectives on ageism (= International perspectives on aging, vol. 19). Cham: Springer, 2018, pp.193–212.

[9] Goriup J. Socialna gerontologija in problem diskurza ageizma. In: Filej B, Kaučič BM, Kristovič S, et al. (eds) Znanost in kultura za zdravo družbo. Maribor: Alma Mater Europaea, 2015, pp.114–119.

[10] Ben-Harush A, Shoivitz-Ezra S, Doron I, et al. Ageism among physicians, nurses and social workers: findings from a qualitative study. Eur J Ageing 2017; 14: 39–48. doi: 10.1007/s10433-016-0389-9 28804393 PMC5550621

[11] Rodgers V and Gilmour J. Shaping student nurses’ attitudes towards older people through learning and experience. Nurs Prax N Z 2011; 27(3): 13–20. 22375376

[12] Kydd A and Fleming A. Ageism and age discrimination in health care: fact or fiction? A narrative review of the literature. Maturitas 2015; 81(4): 432–438. doi: 10.1016/j.maturitas.2015.05.002 26044073

[13] Hyun K and Hansol K. Ageism and Psychological Well-Being Among Older Adults: A Systematic Review. Gerontol Geriatr Med 2022; Jan-Dec: 8. 10.1177/23337214221087023 PMC9008869 35434202

[14] Salway SM, Payne N, Rimmer M, et al. Identifying inequitable healthcare in older people: systematic review of current research practice. Int J Equity Health 2017; 16: 123. doi: 10.1186/s12939-017-0605-z 28697768 PMC5505033

[15] Voss P, Bodner E and Rothermund K. Ageism: the relationship between age stereotypes and age discrimination. Int Perspect Aging 2018; 11–31.

[16] Meisner BA. Physicians’ attitudes toward aging, the aged and the provision of geriatric care: a systematic narrative review. Crit Public Health 2012; 2: 61–72.

[17] Nguyen TT, Vable AM, Glymour MM, et al. Trends for reported discrimination in health care in a national sample of older adults with chronic conditions. J Gen Intern Med 2018; 291–297. doi: 10.1007/s11606-017-4209-5 29247435 PMC5834956

[18] Swift H and Abrams D. The risk of ageism model: how ageism and negative attitudes toward age can be barrier to active aging. Soc Issues Policy Rev 2017; 11(1): 195–231.

[19] Salter B and Salter C. The politics of ageing: health consumers, markets and hegemonic challenge. Sociol Health Illn 2018; 40(6): 1069–1086. doi: 10.1111/1467-9566.12743 29740838

[20] Thompson S. Ageing and ageism. In: Thompson N and Cox GR (eds) Handbook of the sociology of death, grief, and bereavement: a guide to theory and practice. New York: Routledge, 2017, pp.210–223.

[21] Lloyd-Sherlock PG, Ebrahim S, McKee M, et al. Institutional ageism in global health policy. BMJ 2016; 354: 451–454. doi: 10.1136/bmj.i4514 27582131

[22] Liu Y, While AE, Norman IJ, et al. Health professionals’ attitudes toward older people and older patients: a systematic review. J Interprof Care 2012; 397–409. doi: 10.3109/13561820.2012.702146 22780579

[23] Lešnik A and Tomažič J. Starizem v zdravstvenih ustanovah. Obzor Zdr N 2017; 51(4): 312–319.

[24] Skela Savič B, Zurc J and Hvalič Touzery S. Staranje populacije, potrebe starostnikov in nekateri izzivi za zdravstveno nego. Obzor Zdr N 2010; 44(2): 89–100.

[25] Belo Fernandes J, Ramos C, Domingos J, et al. Addressing ageism ‒ be active in aging: study protocol. J Pers Med 2022; 12(3): 354. doi: 10.3390/jpm12030354 35330354 PMC8954157

[26] San-Martin-Gamboa B, Zarazzquin I, Fernandez-Atutxa A, et al. Reducing ageism combining ageing education with clinical practice: a prospective cohort study in health sciences students. Nurs Open 2022; 10: 3854–3861. 10.1002/nop2.1643 PMC10170881 36806648

[27] Podhorechka M, Husejko J, Wozniewicz A, et al. Who will treat older patients? Should medical education focus more on activities aimed at displaying positive attitudes toward older people? The prevalence of ageism among students of medical and health sciences. Front Public Health 2022; 1: 10. 10.3389/fpubh.2022.1032487 PMC9752869 36530671

[28] Burnes D, Shepard C, Henderson CR, et al. Interventions to reduce ageism against older adults: a systematic review and meta-analysis. AJPH 2019; 109(8): e1–e9. doi: 10.2105/AJPH.2019.305123 31219720 PMC6611108

[29] Samra R, Cox T, Lee Gordon A, et al. Factors related to medical students’ and doctors’ attitudes towards older patients: a systematic review. Age Ageing 2017; 46: 911–919. doi: 10.1093/ageing/afx058 28472444 PMC5860378

[30] Mendez A, Lopez M, Rodriguez-Quintanilla K, et al. Ageist no more: interprofessional training for undergraduate healthcare students. Geriatrics 2022; 7(1): 17. doi: 10.3390/geriatrics7010017 35200522 PMC8872434

[31] Nemiroff L. We can do better: addressing ageism against older adults in healthcare. Healthc Manage Forum 2022; 35(2): 118–122. doi: 10.1177/08404704221080882 35199603

