# Supplementary material for: Educator’s role in preventing ageism
Source: PLoS One. 2024 Nov 4;19(11):e0313137. doi: 10.1371/journal.pone.0313137 (PMC11534200; doi:10.1371/journal.pone.0313137)
Supplement: S1 Fig — (PDF) [file pone.0313137.s001.pdf]

Statements about ageism (Questions 1-10)

In the table, mark the opinion on the subject of ageism that comes closest to yours:

Assertion	I do not agree at all	I do not agree	I neither agree nor agree	I agree	I strongly agree
1. Ageism is an important determinant of health.					
2. Ageism manifests itself as a stereotype that reflects thinking about older people					
3. Ageism manifests itself as a prejudice that reflects the perception of older people.					
4. Ageism manifests itself as discrimination that reflects attitudes towards older people.					
5. As the number of older people increases, discriminatory attitudes towards older people are reinforced.					
6. Ageism is deceived.					
7. Ageism is unconscious					
8. Discrimination against older people is not a new					

phenomenon, but it has intensified in recent years.					
9. Ageism also exists in health and social institutions.					
10. Ageism leads to inequality in health care.					

Reasons for ageism (Questions 11-17)

Mark in the table to what extent you agree that each statement influences the occurrence of ageism:

Assertion	I do not agree at all	I do not agree	I neither agree nor agree	I agree	I strongly agree
11. Lack of knowledge.					
12. Anxiety associated with aging.					
13. Age of education participants.					
14. Growing number of older people.					
15. Personal underestimation of older people.					
16. Social underestimation of older people.					
17. Displacement of older people to the margins of society.					

Skills to reduce ageism (Questions 18-26)

Mark in the table to what extent you agree that the listed skills would help to reduce ageism:

Assertion	I do not agree at all	I do not agree	I neither agree nor agree	I agree	I strongly agree
18. Communication with older people.					
19. Knowledge of the needs of older people.					
20. Understanding of the heterogeneity of the older population.					
21. Understanding of the values of health professionals in relation to building a relationship with older people.					
22. Approach towards older people.					
23. Constructive collaboration of health professionals in relation to attitudes towards older people.					
24. Knowledge and respect of roles in interprofessional collaboration.					
25. Knowledge acquired through intergenerational collaboration.					
26. Critical thinking in the context of ageism.					

Ageism among trainees (Questions 27-32)

Mark in the table to what extent the following statements apply to the participants of the training:

Assertion	Never	Rarely	Sometimes	Often	Always
27. The training participants have prejudices (perceptions) about older people.					
28. Training participants have discriminatory attitudes towards older people.					
29. Training participants think stereotypically about older people.					
30. The participants of the training have prejudices (perceptions) about working with older people.					
31. Training participants have discriminatory attitudes about working with older people.					
32. Training participants have stereotypical thinking about working with older people.					

Educational work (Questions 33-38)

Mark in the table to what extent the individual statements apply to your teaching activities:

Assertion	Never	Rarely	Sometimes	Often	Always
33. My curricula are adapted to the aging population trend.					
34. I adapt my curricula to the trends of the aging population.					
35. I transfer examples of good practice from Slovenia and abroad into my teaching methods.					
36. Participants in the training have the opportunity to gain practical experience with older people.					
37. Training participants have the opportunity for intergenerational cooperation.					
38. Training participants have the opportunity to reflect after contact (work) with older people.					

39. How would you rate your knowledge of gerontology and working with older people?

1. very poor
2. poor
3. neither bad nor good
4. good
5. very good

40. What is your attitude to working with older people?

1. I am not at all interested in working with older people.
2. I am not interested in working with older people.
3. I am neither interested nor interested in working with older people.
4. I am interested in working with older people.

5. I am very interested in working with older people.

41. Do you have practical experience of working with older people?
1. yes
2. no
3. I do not know

Demographic data

Title:

1. assistant
2. senior lecturer
3. lecturer
4. docent
5. associate professor
6. full professor
7. university teacher
8. teacher
9. teacher of practical lessons
10. organizer of practical lessons
11. practical training organizer
12. organizer of work practice
13. clinical mentor
14. other:

Indicate the level of education at which you are working:

1. secondary school professional programs
2. higher education professional study programs
3. university study programs
4. master's study programs
5. unified master's study programs
6. doctoral study programs
7. another

Indicate the statistical region in which you are employed:

1. Pomurska region
2. Podravska region
3. Koroška region
4. Savinjska region
5. Zasavska region
6. Posavska region
7. Jugovzhodna region
8. Osrednjeslovenska region
9. Primorsko-notranjska region
10. Goriška region
11. Obalno-kraška region

Write how many years of service you have: ____

Write your age: ____

Please indicate your gender:
1. a man
2. a woman
