# Supplementary material for: Educator’s role in preventing ageism
Source: PLoS One. 2024 Nov 4;19(11):e0313137. doi: 10.1371/journal.pone.0313137 (PMC11534200; doi:10.1371/journal.pone.0313137)
Supplement: S3 Fig — (PDF) [file pone.0313137.s003.pdf]

Construct	Items	Mean	Std. deviation
1: Statements about ageism	Ageism is an important determinant of health.	3.73	1.152
	Ageism manifests itself as a stereotype that reflects thinking about older people	4.23	.742
	Ageism manifests itself as a prejudice that reflects the perception of older people.	4.13	.818
	Ageism manifests itself as discrimination that reflects attitudes towards older people.	4.00	.935
	As the number of older people increases. discriminatory attitudes towards older people are reinforced.	3.30	1.105
	Ageism is deceived.	3.34	.869
	Ageism is unconscious	3.06	.862
	Discrimination against older people is not a new phenomenon. but it has intensified in recent years.	3.70	.878
	Ageism also exists in health and social institutions.	4.02	.788
	Ageism leads to inequality in health care.	4.00	.830
2: Reasons for ageism	Lack of knowledge.	3.92	.834
	Anxiety associated with aging.	3.66	.835
	Age of education participants.	3.31	.968
	Growing number of older people.	3.52	.922
	Personal underestimation of older people.	3.88	.945
	Social underestimation of older people.	4.07	.799
	Displacement of older people to the margins of society.	3.90	.923
3: Skills to reduce ageism	Communication with older people.	4.35	.626
	Knowledge of the needs of older people.	4.47	.677
	Understanding of the heterogeneity of the older population.	4.47	.677
	Understanding of the values of health professionals in relation to building a relationship with older people.	4.31	.748
	Approach towards older people.	4.39	.668

	Constructive collaboration of health professionals in relation to attitudes towards older people.	4.40	.617
	Knowledge and respect of roles in interprofessional collaboration.	4.25	.777
	Knowledge acquired through intergenerational collaboration.	4.42	.601
	Critical thinking in the context of ageism.	4.28	.772
4: Ageism among trainees	The training participants have prejudices (perceptions) about older people.	2.97	.580
	Training participants have discriminatory attitudes towards older people.	2.70	.595
	Training participants think stereotypically about older people.	3.14	.668
	The participants of the training have prejudices (perceptions) about working with older people.	2.92	.735
	Training participants have discriminatory attitudes about working with older people.	2.72	.659
	Training participants have stereotypical thinking about working with older people.	3.10	.778
5: Educational work	My curricula are adapted to the aging population trend.	3.93	.905
	I adapt my curricula to the trends of the aging population.	3.99	.833
	I transfer examples of good practice from Slovenia and abroad into my teaching methods.	3.98	.807
	Participants in the training have the opportunity to gain practical experience with older people.	4.00	.958
	Training participants have the opportunity for intergenerational cooperation.	3.76	.969
	Training participants have the opportunity to reflect after contact (work) with older people.	3.83	.972

Variables	Group	Frequency	Percent
1. Knowledge of gerontology.	Very poor	1	1.1
	Poor	2	2.3
	Neither	18	20.5
	Good	56	63.6
	Very good	11	12.5
	Not interested at all	1	1.1

2. Attitude toward working with older people.	Not interested	2	2.3
	Neither	11	12.5
	Interested	47	53.4
	Very interested	27	30.7
3. Practical experience in working with older people.	Yes	82	93.2
	No	6	6.8

Variables	Parameter	Frequency	Percent
Title	Assistant	13	10.2
	Senior lecturer	9	7.0
	Lecturer	7	5.5
	Docent	7	5.5
	Associate professor	3	2.3
	Full professor	3	2.3
	University teacher	6	4.7
	Teacher	4	3.1
	Teacher of practical lessons	9	7.0
	Organizer of practical lessons	2	1.6
	Practical training organizer	6	4.7
	Organizer of work practice	9	7.0
	Clinical mentor	24	18.8
	Other	26	20.3
Level of education (work)	Secondary school professional programs	4	3.6
	Higher education professional study programs	60	54.5
	University study programs	19	17.3
	Master's study programs	11	10.0
	Unified master's study programs	6	5.5
	Doctoral study programs	10	9.1
	Another	0	0
Statistical region (work)	Pomurska region	4	2.0
	Podravska region	60	30.5
	Koroška region	19	9.6
	Savinjska region	11	5.6
	Zasavska region	6	3.0
	Posavska region	10	5.1
	Jugovzhodna region	1	0.5

	Osrednjeslovenska region	7	3.6
	Primorsko-notranjska region	2	1.0
	Goriška region	4	2.0
	Obalno-kraška region	1	0.5
Gender	Female	76	86.4
	Male	12	13.6
Variable	Mean	Std. deviation	
Years of service	23.66	10.91	
Age	48.02 (mean)	10.60	
